# Retraction: Downregulation of microRNA-142-3p inhibits the aggressive phenotypes of rheumatoid arthritis fibroblast-like synoviocytes through inhibiting Nuclear factor-kappa B signaling

**DOI:** 10.1042/BSR-20190700_RET

**Published:** 2020-09-01

**Keywords:** Inflammation, MicroRNA-142-3p, Nuclear factor-kappa B, Proliferation, Tumor necrosis factor α

This retraction follows an Expression of Concern relating to this article previously published by Portland Press.

This article is being retracted from *Bioscience Reports* at the request of the Editor in Chief and the Editorial Board following receipt of a notification from a reader, alerting the Editorial Board to repeated bands in the Western blots of Figures 1D and E, as well as image manipulation in Figure 4B.

The authors were contacted regarding the concerns raised and provided a replacement Figures 1D and 4B, however given that the original images in Figure 4B showed signs of manipulation, the Editorial Board stands by the decision to retract. The authors disagree to the retraction.

